# Prevalence of Seropositivity to Pandemic Influenza A/H1N1 Virus in the United States following the 2009 Pandemic

**DOI:** 10.1371/journal.pone.0048187

**Published:** 2012-10-31

**Authors:** Carrie Reed, Jacqueline M. Katz, Kathy Hancock, Amanda Balish, Alicia M. Fry, H1N1 Serosurvey Working Group

**Affiliations:** Influenza Division, Centers for Disease Control and Prevention, Atlanta, Georgia, United States of America; University of Hong Kong, Hong Kong

## Abstract

**Background:**

2009 pandemic influenza A/H1N1 (A(H1N1)pdm09) was first detected in the United States in April 2009 and resulted in a global pandemic. We conducted a serologic survey to estimate the cumulative incidence of A(H1N1)pdm09 through the end of 2009 when pandemic activity had waned in the United States.

**Methods:**

We conducted a pair of cross sectional serologic surveys before and after the spring/fall waves of the pandemic for evidence of seropositivity (titer ≥40) using the hemagglutination inhibition (HI) assay. We tested a baseline sample of 1,142 serum specimens from the 2007–2008 National Health and Nutrition Examination Survey (NHANES), and 2,759 serum specimens submitted for routine screening to clinical diagnostic laboratories from ten representative sites.

**Results:**

The age-adjusted prevalence of seropositivity to A(H1N1)pdm09 by year-end 2009 was 36.9% (95%CI: 31.7–42.2%). After adjusting for baseline cross-reactive antibody, pandemic vaccination coverage and the sensitivity/specificity of the HI assay, we estimate that 20.2% (95%CI: 10.1–28.3%) of the population was infected with A(H1N1)pdm09 by December 2009, including 53.3% (95%CI: 39.0–67.1%) of children aged 5–17 years.

**Conclusions:**

By December 2009, approximately one-fifth of the US population, or 61.9 million persons, may have been infected with A(H1N1)pdm09, including around half of school-aged children.

## Introduction

In April 2009, a novel influenza virus was first detected in two children in the United States with a unique combination of genetic sequences not previously detected in animals or humans [1]. The spread of 2009 pandemic influenza A/H1N1 virus [A(H1N1)pdm09] resulted in a pandemic with widespread illness worldwide. In the United States, pandemic activity resulted in a relatively small spring wave with large focal outbreaks in some areas, followed by a larger fall wave of activity which peaked nationwide in late October 2009 and had mostly declined by early 2010 [2].

Serologic surveys can be valuable for determining the incidence of infection caused by a novel virus and making inferences about the level of infection or immunity in a population. Tests such as the hemagglutination-inhibition (HI) assay have long been used to detect serologic responses to influenza virus infection or vaccination. An HI titer of ≥40 is generally associated with reduced susceptibility to influenza infection, and has been used widely as a marker of immunity or past infection with influenza virus [3], [4], [5].

Seasonal influenza viruses generally have small antigenic and genetic changes each year and thus are susceptible to some cross-protective immunity among the population, while A(H1N1)pdm09 was antigenically and genetically distinct from recent human seasonal influenza H1N1 viruses [6]. Because A(H1N1)pdm09 is derived from influenza viruses that had primarily circulated only among swine, little immunity among the general population would be expected. However, A(H1N1)pdm09 is more similar to historical H1N1 viruses that began circulating among humans during the 1918 pandemic and spread into swine populations worldwide [7]. As a result, some degree of baseline cross-reactivity with A(H1N1)pdm09 has been shown to exist in older US adults and international cohorts [8], [9], [10], [11]; however, the frequency of cross-reactive antibody among the general US population before the pandemic began is unclear.

To better characterize population immunity and the incidence of A(H1N1)pdm09 in the United States, we conducted two serologic surveys for the prevalence of A(H1N1)pdm09 antibodies and collected information on pandemic vaccination coverage. The objective was to measure the overall and age-specific increase in seropositivity to A(H1N1)pdm09 by the end of December 2009 (subsequently referred to as year-end 2009) and to estimate the contribution of vaccination and natural infection during the spring and fall US pandemic waves.

## Methods

To determine the increase in seropositivity to A(H1N1)pdm09 by year-end 2009, we conducted cross-sectional serologic surveys before and after the pandemic waves in the United States.

### Pre-pandemic baseline serum specimens

To establish pre-pandemic baseline levels of cross-reactive antibody to A(H1N1)pdm09 by age group, we tested a weighted subsample of banked serum specimens collected during the 2007–2008 National Health and Nutrition Examination Survey (NHANES), an ongoing population-based survey conducted nationwide in the United States which includes extensive health information from both face-to-face interviews and medical examinations. NHANES involves a complex, multistage, probability sampling design to select participants representative of the civilian, non-institutionalized US population. To fully consider the sampling design and the oversampling of certain population subgroups, results were analyzed in SUDAAN using standard survey methods to accommodate the sample weights (WTH1N1) and the sampling frame [12]. All estimates were assessed for design effects, degrees of freedom, and relative standard error. Estimates were not reported if the relative standard error was greater than 30%. The protocol for the use of the NHANES samples was approved by the Research Ethics Review Board of the National Center for Health Statistics (NCHS).

### 2009 serum specimens

To provide a broad geographic representation of the US population following the second wave of the pandemic, we collected the second set of serum specimens during December 2009–January 2010 from ten US sites, representing each of the 10 designated Department of Health and Human Services regions – Connecticut, New York City, Pennsylvania, Florida, Wisconsin, Texas, Missouri, Utah, Arizona, and Washington. Serum specimens included in the 2009 serosurvey were remaining specimens that had been submitted for routine screening from clinical diagnostic laboratories in the included states.

Sites conducted stratified random sampling of eligible specimens within the following age groups: 0–4 years, 5–17 years, 18–24 years, 25–49 years, 50–64 years, 65+ years. Sites were requested to submit 50 specimens per age group, although this sample size was not achievable for all age groups in some sites. In those instances, states sent all specimens available in that age group.

Only data on the patient's age and the date of specimen collection were submitted with each serum specimen. Otherwise, specimens were delinked from all other data and personal identifying information prior to shipping the specimens to CDC.

### Serologic testing

Each serum specimen from the baseline and 2009 samples was tested in triplicate using the hemagglutination-inhibition (HI) assay using 0.5% turkey red blood cells, beta-propriolactone inactived A/California/7/2009 virus and previously described methods [13]. Sera were pre-treated with receptor destroying enzyme (Denke Seiken) according to the manufacturer's recommendations and were used at a starting dilution of 1∶10 with two-fold serial dilutions. A specimen was considered seropositive if the geometric mean HI titer was ≥40, a titer generally associated with reduced susceptibility to infection with influenza virus [3], [4]. We also evaluated a titer cutoff of ≥20, which has been shown in recent studies to be sensitive and specific for detecting PCR-confirmed infection with A(H1N1)pdm09 during the first US wave of the pandemic [14].

### H1N1 vaccination coverage

Because the HI assay does not distinguish between antibody titers elicited by natural infection from those stimulated through vaccination, we also collected information on A(H1N1)pdm09 vaccine coverage for the included states. Weekly pandemic vaccination coverage estimates for each state, stratified by age group, were calculated by CDC using combined Behavioral Risk Factors Surveillance System (BRFSS) and National 2009 H1N1 Flu Survey (NHFS) data, as described previously [15]. Because there is a lag during which persons mount a measurable immune response following vaccination, we applied the vaccination coverage estimate from 2 weeks prior to the date of specimen collection. Using these data, we calculated an overall weighted coverage estimate across states for each age group based on the number of specimens collected per state each week.

### Data and statistical analysis

The HI titer results were used to calculate the overall, state-specific and age-specific prevalence of seropositivity against A(H1N1)pdm09 among the pre-pandemic and 2009 specimens. The increase in seroprevalence from baseline to December 2009 was calculated as the difference in the prevalence of seropositivity to A(H1N1)pdm09 between the 2009 and baseline sera. Because there was insufficient power to calculate increased prevalence of seropositivity stratified by both age and state, results of specimens across states were pooled to calculate age-specific increases in the prevalence of seropositivity from baseline to December 2009. In addition, because so few specimens in the 2007–08 sample were among children age <5 years, baseline results were not able to be calculated for this age group. Based on other serologic studies of H1N1pdm09, we assumed for further analyses that this age group had no baseline seropositivity.

### Estimating the population with natural infection

To estimate the cumulative incidence of infection in the US population given the observed increase in seroprevalence in our study population, we conducted a series of calculations using four equations in a probabilistic analysis using Monte Carlo simulations.

#### Adjusted increase in seroprevalence

Not all persons with virologically-confirmed A(H1N1)pdm09 infection develop HI antibody titers of ≥40. Results of a recent study demonstrated a sensitivity of an HI cutoff ≥40 of 75% among early symptomatic cases who sought outpatient care and had laboratory-confirmed A(H1N1)pdm09 infection; the corresponding specificity was 97% among persons aged <60 years and 94% among persons aged 60+ years [14]. To adjust for the sensitivity and specificity of the HI assay at detecting persons infected with A(H1N1)pdm09, we adjusted the increase in seroprevalence from baseline to December 2009 by age group (see additional detail in appendix S1):
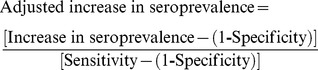



#### Vaccine-related seropositivity

The increase in seroprevalence from baseline to 2009 resulted from both the serologic response to infection with A(H1N1)pdm09 and the vaccine-induced antibody response from A(H1N1)pdm09 vaccination. To identify the contribution of A(H1N1)pdm09 vaccination, which began nationwide in October 2009 and was increasing during the study period, we used data on age-specific vaccine coverage (VC) in the included states and data from published studies on the proportion of individuals that made a serologic response (HI titer ≥40) to vaccination with a 15 µg dose of monovalent, unadjuvanted inactivated A(H1N1)pdm09 vaccine [16], [17], [18].
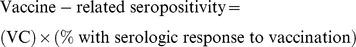



#### Overlap in sources of seropositivity

A person may be seropositive for three reasons – they had baseline cross-reactive antibody prior to the pandemic, they were vaccinated with the pandemic vaccine or they were infected with the pandemic virus. These categories are not mutually exclusive, as some vaccinated persons would have already had pre-existing cross-reactive antibody titers at baseline. Additionally, because vaccine was not widely available until after the peak of pandemic activity in the United States and persons were encouraged to still be vaccinated even if they thought they may have been ill, some vaccinated persons were likely to have been infected with the pandemic virus prior to receiving their vaccination. Because of this possible overlap, if we simply discounted the increase in seropositivity in 2009 due to the entire vaccine coverage, we would underestimate the amount of increase from natural infection.

Because we did not have individual-level data regarding the vaccination status or baseline antibody levels of the persons in our 2009 serosurvey, the exact degree of overlap, or proportion of vaccinated persons with prior seropositivity, is unknown. However, we did identify a range of potential overlap. The minimum overlap would occur if no vaccinated persons had been infected with A(H1N1)pdm09 prior to their vaccination, and thus the only other possible source of seropositivity would be from baseline cross-reactive antibody. We assumed that vaccination status would not be influenced by whether or not someone had pre-existing cross-reactive antibody, thus the minimum proportion of vaccinated persons with prior antibody would be the same as the prevalence of baseline cross-reactive antibody. For example, if 8% of the population had pre-existing cross-reactive antibody, we assumed that 8% of those vaccinated would have also been seropositive from baseline antibody prior to vaccination.

At a maximum, we assumed that vaccinated persons would not be any more likely to have been infected or have pre-existing antibody than the rest of the population. For example, if 20% of the unvaccinated population was seropositive in December 2009, we assumed that at most 20% of those vaccinated could have also been seropositive prior to vaccination. Since we didn't directly measure seropositivity among the unvaccinated, we estimated it for each age group. To calculate this value, we removed vaccine-related seropositivity and calculated the remaining prevalence of seropositivity (further explanation is found in appendix S1):
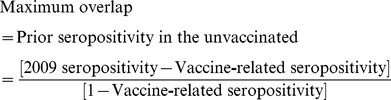



#### Cumulative incidence

Finally, the estimated cumulative incidence of infection in the population was calculated by subtracting the vaccine-related seropositivity from the observed increase in seropositivity and adding back the estimated proportion of the overlap in the vaccinated population who may have had prior seropositivity:
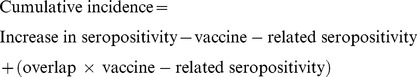
To consider the variability in the estimates used in these calculations, the series of calculations described above were performed by age group in a probabilistic analysis using Monte Carlo simulations with 1,000 iterations. For values of baseline and 2009 seroprevalence and vaccine coverage, normal distributions were fit to the point estimates and their standard error and values were sampled at random from the distribution. Because there was no additional data on the distribution of the overlap between vaccination and prior seropositivity, we selected values at random from a uniform distribution between the minimum and maximum values described previously. All calculations were performed by age group, with the median and 95% probability limits reported based on the distribution of results from the Monte Carlo simulations. All analyses were performed in SAS version 9.2 (SAS Institute, Cary, NC).

## Results

**Figure 1:**
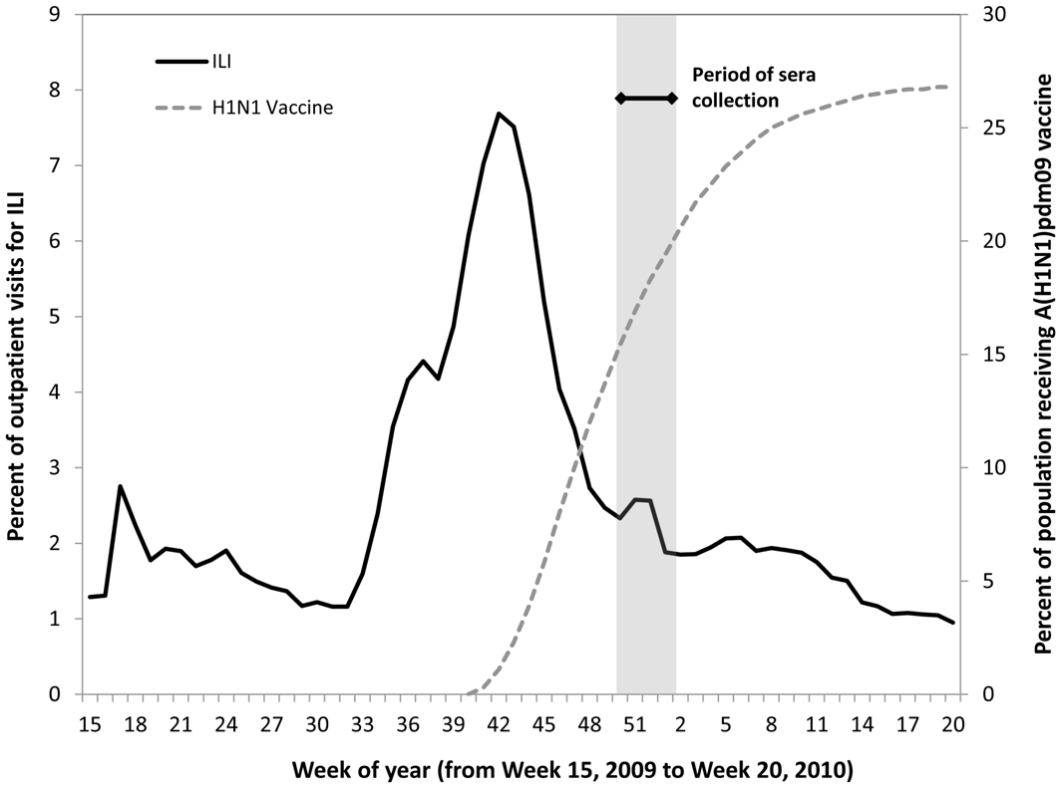
National trends in influenza–like illness (ILI) (solid line)^a^ and coverage with A(H1N1)pdm09 vaccine (dashed line)^b^ in the United States, and the period of sera collection. ^a^ Source: U.S. Outpatient Influenza-like Illness Surveillance Network (ILINet). ^b^ Source: CDC estimates from combined Behavioral Risk Factors Surveillance System (BRFSS) and National 2009 H1N1 Flu Survey (NHFS) data.

A total of 1,142 baseline serum specimens were obtained from the 2007–08 NHANES sera collection and 2,759 serum specimens were obtained from laboratories in 10 states. The volumes of one baseline and four 2009 specimens were not sufficient for HI testing and were excluded. The 2009 specimens were collected from the patients during December 14, 2009–January 15, 2010. Because it may take approximately 2 weeks to mount a measurable immune response following infection or vaccination, we assumed this period of specimen collection corresponded to exposure as of December 1–December 31, 2009. The peak of influenza-like illness (ILI) activity from nationwide surveillance of sentinel outpatient providers had occurred several weeks prior to the collection of study specimens (Figure 1). During the period of sera collection, ILI activity was low and still decreasing, while A(H1N1)pdm09 vaccination coverage was increasing. ILI activity by state for the 10 study states is included as a supplemental figure (Figure S1).

**Figure 2:**
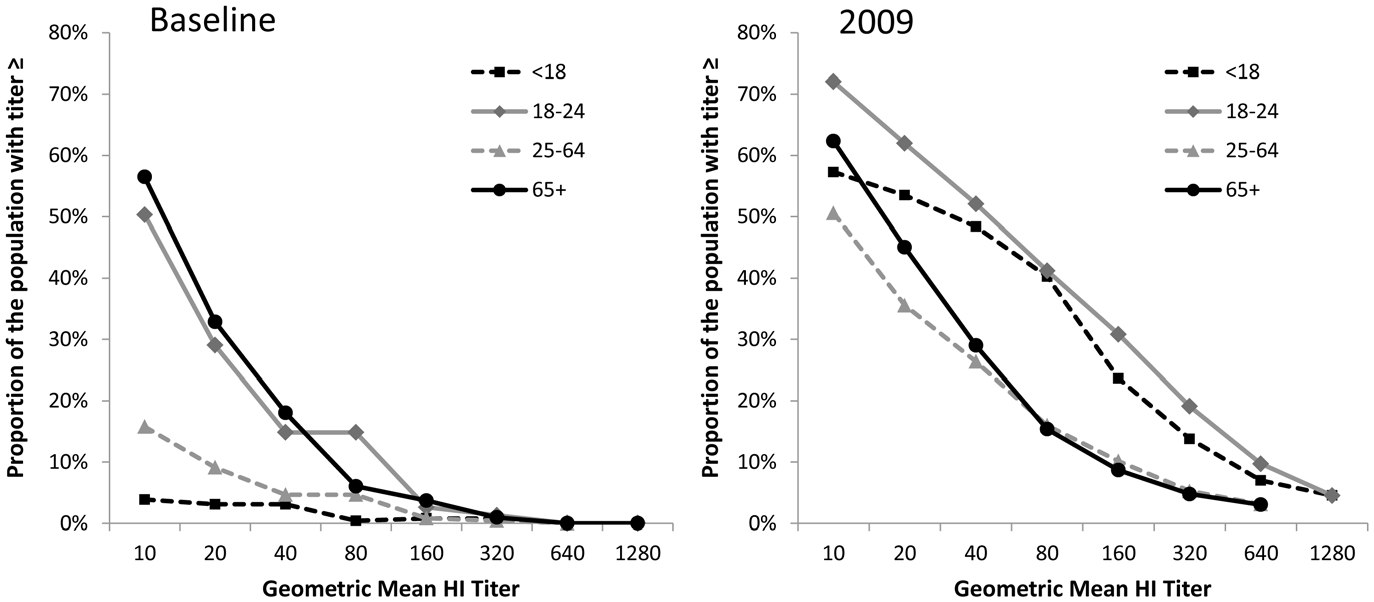
Comparison of the reverse cumulative distribution of HI titers between baseline and 2009 serum specimens, by age group.

The prevalence of seropositivity to A(H1N1)pdm09 among baseline and 2009 serum specimens is presented in Table 1. Because so few specimens in the 2007–08 sample were among children age <5 years, baseline results were not presented for this age group and were assumed to have no baseline cross-reactive antibody. In addition, results from age groups 25–49 and 50–64 years were very similar for all analyses, thus these age groups were combined to maintain sufficient precision. Figure 2 shows age-standardized reverse cumulative distribution curves for the HI titers of individuals included in the baseline and 2009 surveys. The distribution of HI titers in the sampled populations was substantially shifted upward for the 2009 specimens compared to the baseline specimens.

**Table 1:** Overall prevalence of seropositivity to A(H1N1)pdm09 at baseline and December 2009, by age group.

	Baseline		2009		
Age group	No. of samples	% positive^a^(95%CI)	No. of samples	% positive(95%CI)	Difference above baseline(95%CI)
**HI titer ≥40**					
<5 years	45	—	325	36.9 (31.7–42.2)	36.9 (31.7–42.2)^b^
5–17 years	273	9.5 (4.8–14.2)	500	62.2 (57.9–66.5)	52.7 (46.6–58.8)
18–24 years	95	18.5 (7.7–29.3)	454	44.7 (40.1–49.3)	26.2 (15.4–37.0)
25–64 yrs	511	6.8 (2.6–11.7)	963	26.2 (15.4–37.0)	19.6 (15.7–23.4)
65+ yrs	217	15.7 (8.3–23.1)	513	28.3 (24.4–32.2)	12.6 (4.7–20.4)
**TOTAL** c	**1141**	**9.2 (7.1–11.3)**	**2755**	**35.4 (33.6–37.2)**	**26.2 (23.5–28.8)**
**HI titer ≥20**					
<5 years	45	—	325	41.2 (35.9–46.6)	41.2 (35.9–46.6)
5–17 years	273	15.2 (10.0–20.5)	500	69.8 (65.8–73.8)	54.6 (48.3–60.9)
18–24 years	95	27.5 (12.5–42.4)	454	55.3 (50.7–59.9)	27.8 (13.5–42.1)
25–64 yrs	511	14.3 (8.4–19.4)	963	35.1 (32.1–38.1)	20.8 (16.3–25.4)
65+ yrs	217	34.0 (24.9–43.0)	513	43.3 (39.0–47.6)	9.3 (0.1–18.7)
**TOTAL** c	**1141**	**17.5 (15.8–19.1)**	**2755**	**44.6 (42.7–46.5)**	**27.1 (24.8–29.5)**

a Estimates were weighted using the adjusted sample weight for this sample, WTH1N1.

b Assuming no pre-existing seropositivity at baseline.

c Age-standardized to the U.S. Census Bureau population estimates as of July 1, 2009 by age groups in the table.

With an HI titer cutoff of ≥40, the age-adjusted increase in seroprevalence to A(H1N1)pdm09 from baseline to December 2009 was 26% (95%CI: 24–29%), with the highest increase in seroprevalence in those aged 5–17 years (55%, 95%CI: 48–61%). We also considered an HI titer cutoff of ≥20, which has been shown to be more sensitive for A(H1N1)pdm09 infection [14], however the increase from baseline was similar to that obtained with a cut-off titer of ≥40. Thus all further calculations were conducted using a seropositivity cutoff of ≥40, which has been shown to be more specific for A(H1N1)pdm09 infection [14] and is recognized as a predictor of influenza immunity in populations [3], [4].

**Figure 3:**
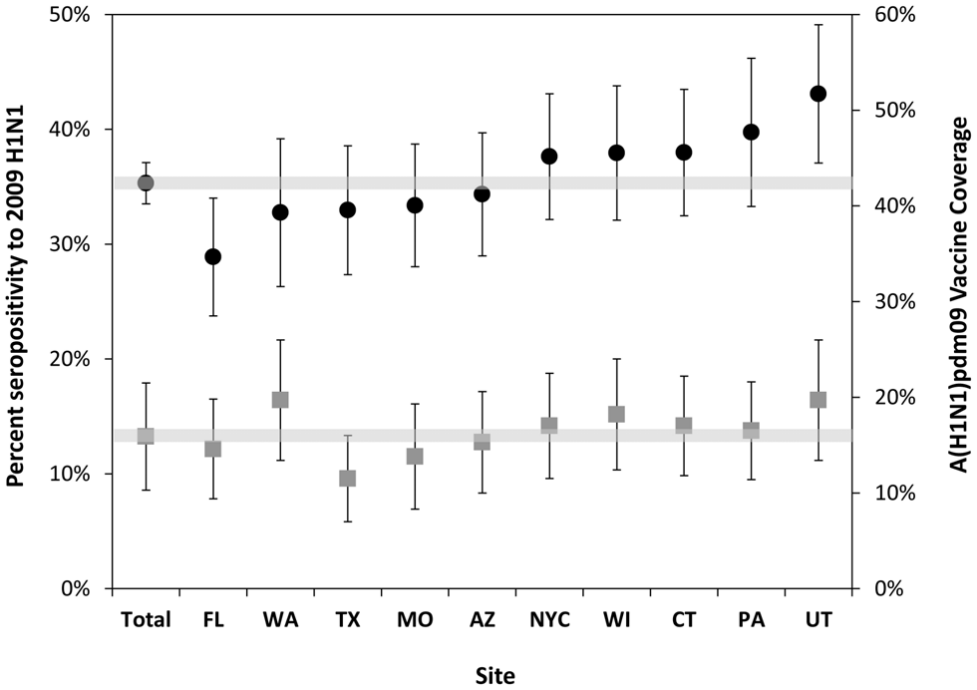
Geographic variation in prevalence of seropositivity to A(H1N1)pdm09 in 2009 (circles) and the proportion of the population reporting A(H1N1)pdm09 vaccination (squares) during the same time period, by state with 95% confidence intervals. Values are age–standardized to the US population.

When stratified by state, the seroprevalence among most states in 2009 was noted to be similar to the overall seroprevalence of 35.4%. There was some geographic variation, notably lower levels of seropositivity in Florida and higher levels in Utah (Figure 3). We also include in Figure 3 the reported vaccine coverage by state during the same time. Not all of the geographic variation in seropositivity was associated with geographic differences in A(H1N1)pdm09 vaccine coverage (Pearson correlation coefficient = 0.56, p = 0.09).

**Figure 4:**
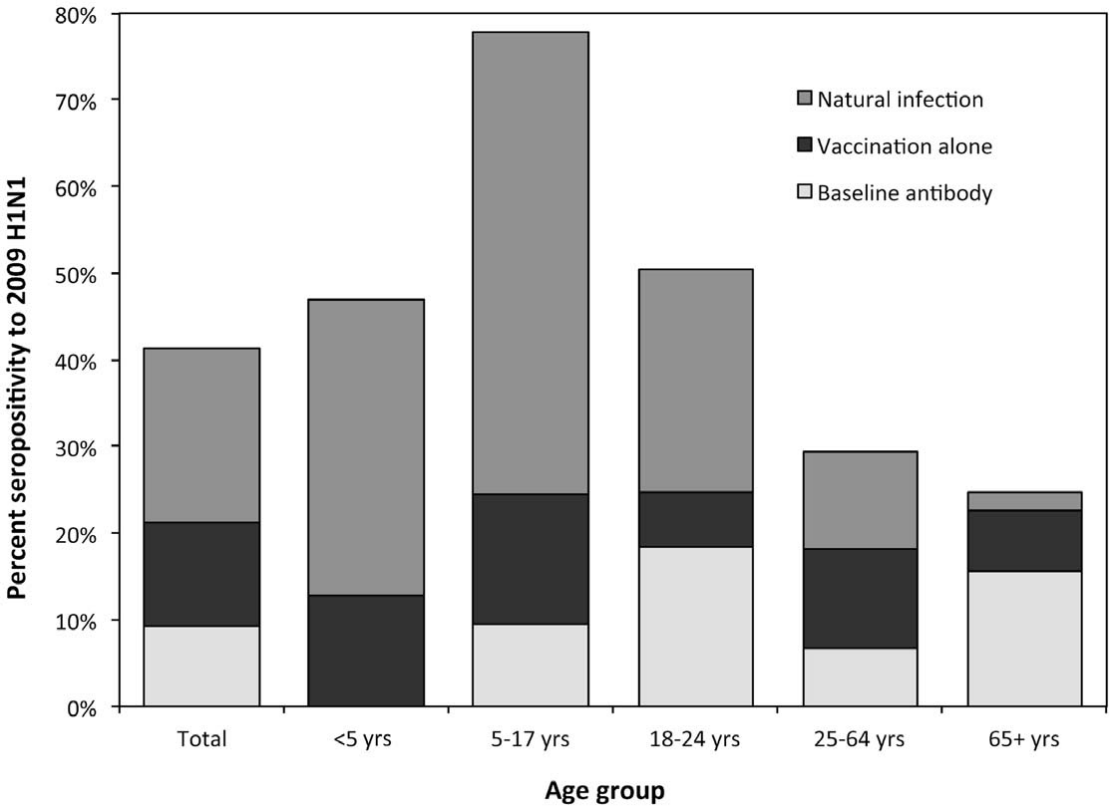
Estimated seroprevalence to A(H1N1)pdm09 through December 2009 by source and age group, adjusted for the sensitivity and specificity of HI assay.

To consider the impact of vaccination and baseline cross-reactive antibody on the December 2009 seroprevalence for A(H1N1)pdm09 and the sensitivity and specificity of the assay, the December 2009 seroprevalence by age group was adjusted to estimate the incidence of A(H1N1)pdm09 infection in 2009. Following adjustment, we estimated that the age-standardized incidence of A(H1N1)pdm09 infection in the population was 20.2% (95%CI: 10.1–28.3%) by year-end 2009 (Table 2), with the highest incidence among children aged 5–17 years (53.3%, 95%CI: 39.0–62.7%). If the aggregate age standardized incidence among the 10 sampled states were representative of the incidence in the entire US population, an estimated total of 61.9 million persons would have been infected in the United States (95%CI: 30.9–86.7) by year-end 2009. The estimated proportion of seropositivity from baseline cross-reactive antibody, pandemic vaccination, and natural infection in the population varied widely between age groups (Figure 4).

**Table 2:** Vaccination and adjusted estimates of the increase in seropositivity from baseline to December 2009 due to natural infection with A(H1N1) pdm2009.

	Vaccine Coverage	Serologic response to vaccination	Estimated seropositivity due to vaccination alone^b^		Estimated incidence of natural infection, 2009^c^	
	% ± SE	%	%	95% CI	%	95% CI
**Total** a	**–**	**–**	**11.9**	4.2–20.8	**20.2**	10.1–28.3
<5 yrs	29.6±9.3	70	**12.8**	4.4–22.7	**34.2**	22.6–45.6
5–17 yrs	25.3±5.8	97	**15.0**	6.7–26.4	**53.3**	39.0–67.1
18–24 yrs	10.9±7.7	97	**6.1**	−2.0–17.5	**25.9**	6.8–42.3
25–64 yrs	14.5±4.7	97	**11.3**	4.1–20.2	**11.3**	1.8–20.4
65+ yrs	11.2±4.6	80	**7.0**	1.0–13.6	**1.9**	−10.0–15.5

SE = Standard error, CI = Confidence interval.

a Age-standardized to the US population.

b Adjusting for overlap from vaccination among people already infected with A(H1N1)pdm09. See methods for description of adjustment.

c After adjusting for vaccination and assuming the following HI test characteristics: Sensitivity = 75%, Specificity = 97% (for ages <65) or 94% (for ages 65+).

## Discussion

We detected a high level of seropositivity to A(H1N1)pdm09 in the United States by December 2009, following the spring and fall waves of the pandemic. Over one third of the sampled population had HI antibody titers ≥40, a titer associated with reduced susceptibility to influenza virus infection [3], [4]. By December 2009 the population seropositivity was particularly high among school-age children and young adults, with 62% and 45% among those age groups having antibody titers ≥40, respectively. If these data from these ten states are considered broadly representative of the national experience, a substantial proportion of population immunity to A(H1N1)pdm09 followed the second wave of the pandemic in the United States.

When we adjusted for the sensitivity and specificity of the HI assay, baseline cross-reactive antibody in the US population, and A(H1N1)pdm09 vaccine coverage in the included states, we estimated that approximately 20% of the US population may have been infected with A(H1N1)pdm09 by year-end 2009, including around half of school aged children and substantially fewer persons aged ≥65 years. These findings are consistent with clinical and epidemiologic data from 2009, which showed elevated rates of A(H1N1)pdm09 illness among children and frequent school outbreaks during the pandemic with relatively low morbidity rates among older adults [2]. Among persons aged ≥65, the higher prevalence of baseline cross-reactive antibody and the limited increase in seroprevalence observed by the end of 2009 support conclusions about pre-existing protective immunity in older adults, which may have resulted from exposure to H1N1 viruses earlier in life. Interestingly, we also found a higher baseline prevalence of seropositivity in young adults aged 18–24 years. The reasons for this are unclear, though similar findings were seen in data from the UK [10].

Several approaches have been used to estimate the incidence of A(H1N1)pdm09 illness from clinical surveillance in the US, England and Australia [19], [20], [21], [22]. A prior CDC study estimated that ∼55 million illnesses had occurred in the US by December 2009 [23], similar to our estimate of 59 million persons with A(H1N1)pdm09 infection by the same time. Disease burden estimates from clinical surveillance may not fully capture milder infections which do not meet a traditional ILI surveillance definition, and thus can underestimate the true community incidence of influenza as noted with serological surveys previously conducted in the UK [10]. Additionally, asymptomatic influenza infections would not be captured in estimates from clinical surveillance, which have been observed in up to ∼30% of those infected [24], [25], [26]. By detecting laboratory-based evidence of past infection, serological surveys complement the more timely estimates from clinical surveillance and provide a fuller measure of the disease burden of A(H1N1)pdm09 virus infection in the population over time.

Prior serosurveys following the second wave of the pandemic have been conducted in several parts of the world, but only in limited geographic regions of the United States – Pittsburgh, Pennsylvania in November 2009 [27] and Tampa Bay, Florida in December 2009 [28]. Both involved populations sampled from different sources than our 2009 serosurvey and found results similar to our seroprevalence estimates. The Pittsburgh investigators detected an overall seroprevalence against A(H1N1)pdm09 of 21% at the end of November 2009 while activity was still decreasing and vaccination was increasing, compared to 39% one month later in our study. The Tampa Bay investigators detected an overall seroprevalence of 25% compared to 29% for Florida in our serosurvey at approximately the same time. Internationally, other serosurveys were conducted at varying time points during the pandemic with results demonstrating similar distributions of infection by age group, notably the highest incidence in school-age children and the lowest increase among the elderly [5], [10], [29], [30], [31]. We did find higher estimates of infection in school-age children than in similar serosurveys from other countries, which ranged generally from 34–43% [32]. The reasons for this difference are not entirely clear, as direct comparison from study to study is difficult due to differences in sampling and design. However, many of the large early outbreaks in the US were in schools and summer camps [2] and the timing of the US fall wave was associated with the start of the school year in August/September [33], suggesting that perhaps some of our higher estimate in children may be a real difference. In addition, however, H1N1pdm09 vaccine was also often administered through school-based vaccination clinics, and our indirect method of accounting for vaccination may not have captured the full impact on our serologic results.

Although some A(H1N1)pdm09 activity continued at low levels during early 2010, no immediate third wave of A(H1N1)pdm09 activity was seen nationally during that time when influenza activity usually peaks in the US. This may be partially explained by the year-end 2009 prevalence of seropositivity detected across age groups, which suggested a large third wave of predominantly A(H1N1)pdm09 infection would be unlikely. However, high levels of seropositivity were likely not geographically homogenous. Even within our sample we saw some geographic variability in seroprevalence, as has also been seen in other countries [10], [32]. Because of limitations in sample size, we pooled specimens across geographic areas to estimate an average prevalence of seropositivity, though especially in some parts of the country many persons likely remained susceptible to future infection. As was seen during the 2010–11 influenza season, circulation of A(H1N1)pdm09 did continue in parts of the United States, with co-circulation of influenza A/H3N2 and influenza B predominating in some regions [34].

Our study was subject to some limitations. Our study consists of two separate cross-sectional serosurveys involving serum collections before and after the pandemic waves to measure the increase in population seropositivity, which is less precise than a serial serosurvey involving multiple specimens from the same individuals. In addition, the 2009 sera were collected from remaining specimens submitted for routine screening to clinical diagnostic laboratories representing each of the included states. This has been a common study design where random population samples of sera are not readily available [32]. Nonetheless, it remains unclear how the sample population may differ from the general US population in probability of A(H1N1) pdm09 infection or vaccination, and thus what biases could be present. Of note, results from other countries' serosurveys based on convenience samples of residual sera did not differ substantially from those serosurveys using systematic sampling from existing cohorts [35].

Finally, because we did not have information regarding vaccination history from included individuals, we made adjustments to the 2009 seroprevalence based on state-level A(H1N1)pdm09 vaccination coverage. Estimates of A(H1N1)pdm09 vaccination coverage were derived from surveys of self-reported vaccination status. While the vaccine questions in these national surveys have undergone cognitive testing, some persons may have confused seasonal and A(H1N1)pdm09 vaccinations, both of which were occurring in the fall of 2009. If we overestimated A(H1N1)pdm09 vaccine coverage, we may have underestimated the proportion of seropositivity resulting from natural infection. Again, however, our estimates after adjustment for vaccination compare similarly to other countries such as New Zealand and Australia that collected sera before vaccine was widely available [5], [32], [36].

Estimates of influenza burden from clinical surveillance are timely, but may not fully capture the burden of infection associated with A(H1N1)pdm09 in the population. Serosurveys may compliment these estimates by providing laboratory evidence of influenza virus infection and immunity among populations to help better characterize disease transmission and population immunity. We found a high level of seropositivity to A(H1N1)pdm09 in the United States by December 2009 after the spring and fall waves of the pandemic, during which we estimated that approximately 20% of the US population may have been infected with A(H1N1)pdm09, including around half of school-age children.

## Supporting Information

Appendix S1 **Supplemental information.**
(DOC)

Figure S1 **Site–specific trends in influenza–like illness (ILI), with period of sera collection.** ^a^ Source: U.S. Outpatient Influenza-like Illness Surveillance Network (ILINet).
(JPG)

## References

[1] Dawood FS, Jain S, Finelli L, Shaw MW, Lindstrom S, et al (2009) Emergence of a novel swine-origin influenza A (H1N1) virus in humans. N Engl J Med 360: 2605–2615. 19423869 10.1056/NEJMoa0903810

[2] Jhung MA, Swerdlow D, Olsen SJ, Jernigan D, Biggerstaff M, et al (2011) Epidemiology of 2009 pandemic influenza A (H1N1) in the United States. Clin Infect Dis 52 Suppl 1: S13–26. 21342884 10.1093/cid/ciq008

[3] de Jong JC, Palache AM, Beyer WE, Rimmelzwaan GF, Boon AC, et al (2003) Haemagglutination-inhibiting antibody to influenza virus. Dev Biol (Basel) 115: 63–73. 15088777

[4] Hobson D, Curry RL, Beare AS, Ward-Gardner A (1972) The role of serum haemagglutination-inhibiting antibody in protection against challenge infection with influenza A2 and B viruses. J Hyg (Lond) 70: 767–777. 4509641 10.1017/s0022172400022610 PMC2130285

[5] Bandaranayake D, Huang QS, Bissielo A, Wood T, Mackereth G, et al (2010) Risk factors and immunity in a nationally representative population following the 2009 influenza A(H1N1) pandemic. PLoS One 5: e13211. 20976224 10.1371/journal.pone.0013211 PMC2954793

[6] Garten RJ, Davis CT, Russell CA, Shu B, Lindstrom S, et al (2009) Antigenic and genetic characteristics of swine-origin 2009 A(H1N1) influenza viruses circulating in humans. Science 325: 197–201. 19465683 10.1126/science.1176225 PMC3250984

[7] Xu R, Ekiert DC, Krause JC, Hai R, Crowe JE Jr, et al (2010) Structural basis of preexisting immunity to the 2009 H1N1 pandemic influenza virus. Science 328: 357–360. 20339031 10.1126/science.1186430 PMC2897825

[8] Hancock K, Veguilla V, Lu X, Zhong W, Butler EN, et al (2009) Cross-reactive antibody responses to the 2009 pandemic H1N1 influenza virus. N Engl J Med 361: 1945–1952. 19745214 10.1056/NEJMoa0906453

[9] Ikonen N, Strengell M, Kinnunen L, Osterlund P, Pirhonen J, et al (2010) High frequency of cross-reacting antibodies against 2009 pandemic influenza A(H1N1) virus among the elderly in Finland. Euro Surveill 15. 20144443

[10] Miller E, Hoschler K, Hardelid P, Stanford E, Andrews N, et al (2010) Incidence of 2009 pandemic influenza A H1N1 infection in England: a cross-sectional serological study. Lancet 375: 1100–1108. 20096450 10.1016/S0140-6736(09)62126-7

[11] Rizzo C, Rota MC, Bella A, Alfonsi V, Declich S, et al (2010) Cross-reactive antibody responses to the 2009 A/H1N1v influenza virus in the Italian population in the pre-pandemic period. Vaccine 28: 3558–3562. 20307592 10.1016/j.vaccine.2010.03.006

[12] NCHS. Continuous NHANES Web Tutorial. Available: http://www.cdc.gov/nchs/tutorials/Nhanes/index_current.htm. Accessed 2011 Jul 13.

[13] WHO. World Health Organization Global Influenza Surveillance Network. Manual for the laboratory diagnosis and virological surveillance of influenza. Available: http://www.who.int/influenza/resources/documents/manual_diagnosis_surveillance_influenza/en/. Accessed 2011 Sept 29.

[14] Veguilla V, Hancock K, Schiffer J, Gargiullo PM, Lu X, et al (2011) Sensitivity and Specificity of Serologic Assays for Detection of Human Infection with 2009 Pandemic H1N1 Virus in U.S. Populations. J Clin Microbiol 49: 2210–2215. 21471339 10.1128/JCM.00229-11 PMC3122722

[15] CDC. FluVaxView, 2009–10 Influenza Season. Available: http://www.cdc.gov/flu/professionals/vaccination/vaccinecoverage.htm#0910. Accessed 2012 Jul 13.

[16] Nolan T, McVernon J, Skeljo M, Richmond P, Wadia U, et al (2010) Immunogenicity of a monovalent 2009 influenza A(H1N1) vaccine in infants and children: a randomized trial. JAMA 303: 37–46. 20026597 10.1001/jama.2009.1911

[17] Plennevaux E, Sheldon E, Blatter M, Reeves-Hoche MK, Denis M (2010) Immune response after a single vaccination against 2009 influenza A H1N1 in USA: a preliminary report of two randomised controlled phase 2 trials. Lancet 375: 41–48. 20018365 10.1016/S0140-6736(09)62026-2

[18] Zhu FC, Wang H, Fang HH, Yang JG, Lin XJ, et al (2009) A novel influenza A (H1N1) vaccine in various age groups. N Engl J Med 361: 2414–2423. 19846844 10.1056/NEJMoa0908535

[19] Baguelin M, Hoschler K, Stanford E, Waight P, Hardelid P, et al (2011) Age-specific incidence of A/H1N1 2009 influenza infection in England from sequential antibody prevalence data using likelihood-based estimation. PLoS One 6: e17074. 21373639 10.1371/journal.pone.0017074 PMC3044152

[20] Dawood FS, Hope KG, Durrheim DN, Givney R, Fry AM, et al (2010) Estimating the disease burden of pandemic (H1N1) 2009 virus infection in Hunter New England, Northern New South Wales, Australia, 2009. PLoS One 5: e9880. 20360868 10.1371/journal.pone.0009880 PMC2848017

[21] Reed C, Angulo FJ, Swerdlow DL, Lipsitch M, Meltzer MI, et al (2009) Estimates of the prevalence of pandemic (H1N1) 2009, United States, April–July 2009. Emerg Infect Dis 15: 2004–2007. 19961687 10.3201/eid1512.091413 PMC3375879

[22] Shrestha SS, Swerdlow DL, Borse RH, Prabhu VS, Finelli L, et al (2011) Estimating the burden of 2009 pandemic influenza A (H1N1) in the United States (April 2009–April 2010). Clin Infect Dis 52 Suppl 1: S75–82. 21342903 10.1093/cid/ciq012

[23] CDC. CDC Estimates of 2009 H1N1 Influenza Cases, Hospitalizations and Deaths in the United States, April–December 12, 2009. Available: http://www.cdc.gov/h1n1flu/estimates/April_December_12.htm. Accessed 2012 Jul 13.

[24] Jackson ML, France AM, Hancock K, Lu X, Veguilla V, et al (2011) Serologically confirmed household transmission of 2009 pandemic influenza A (H1N1) virus during the first pandemic wave–New York City, April–May 2009. Clin Infect Dis 53: 455–462. 21844028 10.1093/cid/cir437

[25] Carrat F, Vergu E, Ferguson NM, Lemaitre M, Cauchemez S, et al (2008) Time lines of infection and disease in human influenza: a review of volunteer challenge studies. Am J Epidemiol 167: 775–785. 18230677 10.1093/aje/kwm375

[26] Papenburg J, Baz M, Hamelin ME, Rheaume C, Carbonneau J, et al (2010) Household transmission of the 2009 pandemic A/H1N1 influenza virus: elevated laboratory-confirmed secondary attack rates and evidence of asymptomatic infections. Clin Infect Dis 51: 1033–1041. 20887206 10.1086/656582

[27] Zimmer SM, Crevar CJ, Carter DM, Stark JH, Giles BM, et al (2010) Seroprevalence following the second wave of Pandemic 2009 H1N1 influenza in Pittsburgh, PA, USA. PLoS One 5: e11601. 20644650 10.1371/journal.pone.0011601 PMC2904390

[28] Cox CM, Goodin K, Fisher E, Dawood FS, Hamilton JJ, et al (2011) Prevalence of 2009 Pandemic Influenza A (H1N1) Virus Antibodies, Tampa Bay Florida — November–December, 2009. PLoS One 6: e29301. 22206008 10.1371/journal.pone.0029301 PMC3243696

[29] Chen MI, Lee VJ, Lim WY, Barr IG, Lin RT, et al (2010) 2009 influenza A(H1N1) seroconversion rates and risk factors among distinct adult cohorts in Singapore. JAMA 303: 1383–1391. 20388894 10.1001/jama.2010.404

[30] Xu C, Bai T, Iuliano AD, Wang M, Yang L, et al (2011) The seroprevalence of pandemic influenza H1N1 (2009) virus in China. PLoS One 6: e17919. 21533034 10.1371/journal.pone.0017919 PMC3080876

[31] Skowronski DM, Hottes TS, Janjua NZ, Purych D, Sabaiduc S, et al (2010) Prevalence of seroprotection against the pandemic (H1N1) virus after the 2009 pandemic. CMAJ 182: 1851–1856. 20956500 10.1503/cmaj.100910 PMC2988533

[32] Kelly H, Peck HA, Laurie KL, Wu P, Nishiura H, et al (2011) The age-specific cumulative incidence of infection with pandemic influenza H1N1 2009 was similar in various countries prior to vaccination. PLoS One 6: e21828. 21850217 10.1371/journal.pone.0021828 PMC3151238

[33] Chao DL, Halloran ME, Longini IM Jr (2010) School opening dates predict pandemic influenza A(H1N1) outbreaks in the United States. J Infect Dis 202: 877–880. 20704486 10.1086/655810 PMC2939723

[34] CDC (2011) Update: influenza activity — United States, 2010–11 season, and composition of the 2011–12 influenza vaccine. MMWR Morb Mortal Wkly Rep 60: 705–712. 21637185

[35] Wu JT, Ma ES, Lee CK, Chu DK, Ho PL, et al (2010) The infection attack rate and severity of 2009 pandemic H1N1 influenza in Hong Kong. Clin Infect Dis 51: 1184–1191. 20964521 10.1086/656740 PMC3034199

[36] Gilbert GL, Cretikos MA, Hueston L, Doukas G, O'Toole B, et al (2010) Influenza A (H1N1) 2009 antibodies in residents of New South Wales, Australia, after the first pandemic wave in the 2009 southern hemisphere winter. PLoS One 5: e12562. 20830210 10.1371/journal.pone.0012562 PMC2935357

