# Supplementary material for: Prevalence of Seropositivity to Pandemic Influenza A/H1N1 Virus in the United States following the 2009 Pandemic
Source: PLoS One. 2012 Oct 31;7(10):e48187. doi: 10.1371/journal.pone.0048187 (PMC3485186; doi:10.1371/journal.pone.0048187)
Supplement: Appendix S1 — Supplemental information. (DOC) [file pone.0048187.s001.doc]

**Prevalence of seropositivity to pandemic influenza A/H1N1 virus in the United States following the 2009 pandemic**

**Appendix – Supplemental information**

Derivation of formulas used to calculate the estimated incidence of infection with 2009 H1N1.

***Adjusted increase in seroprevalence***. We adjusted the increase in measured seroprevalence between baseline and year-end 2009 to account for the sensitivity and specificity of the HI assay. To illustrate this adjustment, refer to the table below, where:

a = increase in seropositivity, as measured by HI assay

s = sensitivity of HI assay

p = specificity of HI assay

|  | True positive | True negative | Total |
| --- | --- | --- | --- |
| Seropositive | s*x | (1-p)*(N-x) | a*N |
| Seronegative | (1-s)*x | p*(N-x) | (1-a)*N |
| Total | x | N-x | N |

Goal = to determine the adjusted increase in seroprevalence after accounting for the sensitivity and specificity of the HI assay, or x/N:

s*x + (1-p)*(N-x) = a*N

s*x + (1-p)*N – (1-p)*x = a*N

s*x – (1-p)*x = a*N – (1-p)*N

x*(s-(1-p) = N*(a-(1-p))

x/N = [a-(1-p)]/[s-(1-p)]

or

Adjusted increase in seroprevalence =

[Increase in seroprevalence – (1-Specificity)]/[(Sensitivity – (1-Specificity)]

***Maximum estimate of prior antibody in vaccinated persons***. We recognized that a fraction of persons who were vaccinated against A(H1N1)pdm09 in the fall of 2009 may have already been seropositive before vaccination, either from having baseline cross-reactive antibody or having already been infected with the virus earlier in the year.

For example, in a population of 100 persons, assume 40 persons were seropositive for A(H1N1)pdm09, giving an estimated prevalence of seropositivity in this population of 40%. If the vaccine coverage was 20%, and 90% of those vaccinated developed seropositivity, then the vaccine-related seropositivity was 18%, or 18 persons could have developed seropositivity due to vaccination. This leaves 82 persons (100-18) who could not have developed seropositivity from vaccination, and among them remained 22 persons (40-18) who were seropositive by the HI assay. Thus, the seropositivity among those not effectively vaccinated was 22/82 or 26.8% from a source other than vaccination (either baseline cross-reactive antibody or prior infection).

For our calculations we assumed that, at a maximum, vaccinated persons would not be any more likely to have been infected or have pre-existing antibody than the rest of the population. Thus, if 26.8% of those not effectively vaccinated had seropositivity to A(H1N1)pdm09 by year-end 2009, then up to 26.8% of those vaccinated persons (26.8% of 18 persons = 4.8 persons) may have been seropositive before they were vaccinated.

Maximum overlap = Seropositivity among those not effectively vaccinated =

[2009 seropositivity – Vaccine-related seropositivity] / [1 – Vaccine-related seropositivity]
