# Supplementary figures and images for: Prevalence of Seropositivity to Pandemic Influenza A/H1N1 Virus in the United States following the 2009 Pandemic
Source: PLoS One. 2012 Oct 31;7(10):e48187. doi: 10.1371/journal.pone.0048187 (PMC3485186; doi:10.1371/journal.pone.0048187)

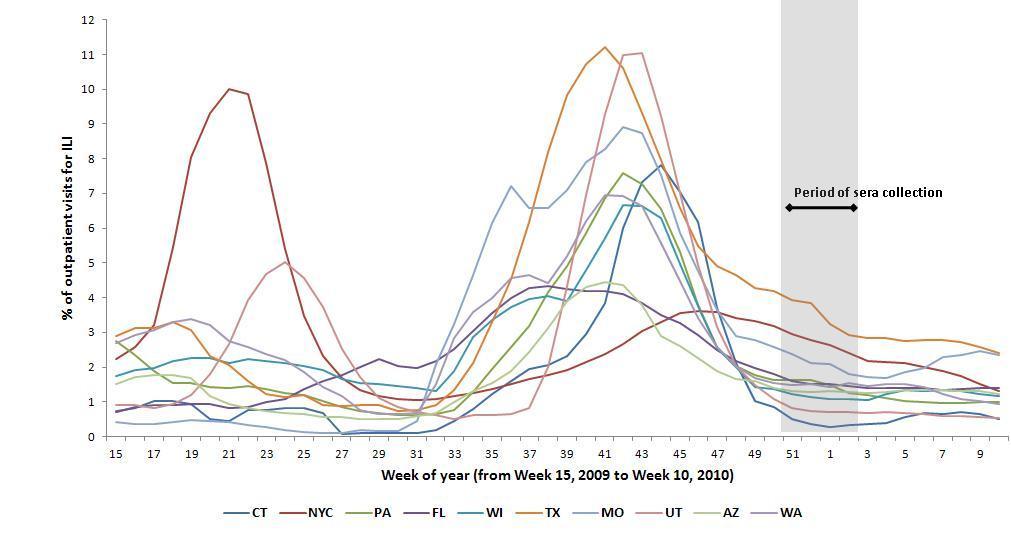

Supplement: Figure S1 — Site–specific trends in influenza–like illness (ILI), with period of sera collection. a Source: U.S. Outpatient Influenza-like Illness Surveillance Network (ILINet). (JPG) [file pone.0048187.s002.jpg]
